# Field investigation and response to a vaccine-derived poliovirus pre-tOPV switch in Southwest Nigeria, October 2015

**DOI:** 10.11604/pamj.2020.37.6.17344

**Published:** 2020-09-02

**Authors:** Akinola Fatiregun, Ekundare Famiyesin, Samuel Bawa, Ninilola Ogunbodede

**Affiliations:** 1 World Health Organization, Ibadan, Nigeria

**Keywords:** Acute flaccid paralysis, vaccine-derived polio virus, oral polio vaccine

## Abstract

A vaccine-derived poliovirus (VDPV) was isolated in an acute flaccid paralysis (AFP) case reported from Ile-Ife, in Osun state, Southwest Nigeria. We investigated the epidemiological characteristics of the polio event and described the immediate public health response that followed. We interviewed the primary caregiver of the case and conducted active case searches for additional AFP cases in the communities in Ife East Local Government Area (LGA) of Osun state. Stool samples of contacts and non-contacts were collected and sent for laboratory investigation. A public health response with mass supplementary immunization in the affected areas followed immediately in the ward the case was located in October 2015. Also, we reviewed the administrative record of the oral polio vaccine (OPV) coverage in the LGA in the previous four years. The VDPV case was a female, one-month-old child with adequate vaccination history for her age. However, the environment of the child was relatively filthy with inappropriate facilities. Laboratory reports from contact samples were negative for VDPV or any polio isolates. A missed AFP case was found from active case searches and a high proportion of under-five children was immunized with tOPV. The OPV3 administrative coverage in the LGA peaked in 2014 (103%) and dropped in 2015 (67%). Efforts directed toward improving environmental hygiene in households and improving OPV coverage in subsequent routine and supplementary immunization are suggested.

## Outbreak investigation report

The global commitment to eradicate wild polioviruses (WPVs) and end poliomyelitis has successfully reduced polio incidence by >99% [1] and eradicated one of the three WPV serotypes, WPV2, in 1999 [2]. By late 2015, all except three countries Pakistan, Afghanistan and Nigeria, have interrupted indigenous transmission of WPV1 and no country has reported a case of WPV3 for over two years [3]. Nigeria, though still listed as endemic polio state, had made significant progress in interrupting transmission of WPV since 2012 when the country accounted for more than half of all polio cases worldwide [3]. Despite these successes, there is the need for vigilance and resilience more than ever before to stop transmission of WPV everywhere contemporaneously [4]. The two fundamental cornerstones of the initiative for polio eradication are the mass oral polio vaccine (OPV) immunisation and sensitive poliovirus surveillance [5]. The immunisation involved the use of multiple doses of OPV, particularly the trivalent OPV (tOPV), which contains live, attenuated types 1, 2 and 3 polioviruses. The OPV has many advantages that include ease of administration and efficient induction of mucosal immunity, thereby limiting poliovirus shedding and person-to-person transmission [2]. The surveillance for poliovirus consists of an investigation of acute flaccid paralysis (AFP) and virologic studies of isolates originating from the stool specimen of AFP cases or the environment wastewater samples.

The purpose of the AFP surveillance is to detect reliably areas where poliovirus transmission is occurring or likely to occur and to allow supplementary immunisation to be focused where it is needed. The process of AFP surveillance in Nigeria has been previously described [6]. Despite the advantages of the OPV, it carries the infrequent risks of genetic mutation and recombination with other vaccine serotypes and other enteroviruses. Thereby, causing vaccine-associated paralytic poliomyelitis among OPV recipients and their direct contacts [2] and the emergence of genetically divergent vaccine-derived polioviruses (VDPVs) [7,8]. The VDPVs are identified based on their degree of genetic divergence from the parent OPV viral strain. Strains that are >1% (for types 1 and 3) or >0.6% (for type 2) divergent from the corresponding OPV strain are labelled as VDPVs. These are further classified into three categories; immunodeficiency related (iVDPV), circulating (cVDPV) and ambiguous (aVDPV) vaccine-derived polioviruses. The iVDPVs are a particular case of VDPVs arising from the guts of persons with primary immunodeficiency.

The cVDPV occurs when there is evidence of person-to-person transmission in the community, while the aVDPV is a classification of exclusion when the investigation does not support classification as cVDPV or iVDPV [7,8]. The occurrence of iVDPVs is infrequent with only 111 cases documented worldwide since 1962. Among these, most stopped excretion within six months or died [9]. Almost all reports of persistent iVDPV infections have been from countries with high to intermediate levels of economic development. In Nigeria, outbreaks of VDPV particularly cVDPV have been previously reported in northern states [10] and no case of VDPV was published to our knowledge from the southern part of the country. Although a previous outbreak of WPV has been reported in Osun state [11], one of the states in the southwest zone of the country, however, no VDPV was reported. In October 2015, an isolate of VDPV associated with an AFP case from more ward, a rural community in Ife East Local Government Area (LGA) of the state, was identified. In this paper, we described the epidemiological features of the polio event and provided the immediate public health actions that followed.

**Figure 1:**
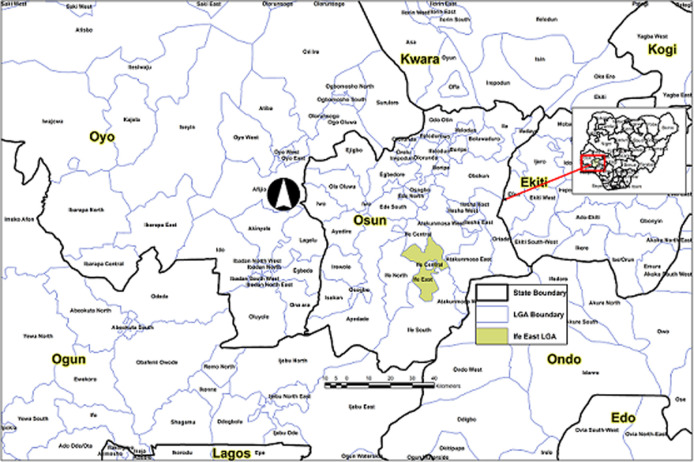
map of Osun state, Nigeria, showing Ife East LGA, including state borders, 2015

**Study setting:** Ife East LGA is one of the 30 LGAs in Osun state that was created in 1996 and one of the 774 LGAs in Nigeria (Figure 1). Administratively, its headquarters is in the town of Oke Ogbo. It has an area of 172km^2^ and a population of 263,661 according to the 2006 population census. It has ten political wards namely; Okerewe 1,2,3; Yekemi; Ilode 1,2; Modakeke 1,2,3; and more. The dominant ethnic group in the LGA is the Yoruba with other minority groups such as Hausa and Igbo. The two major religions practised are christianity and islam, though other traditional religions are recognised. Most of the people are either farmers or civil servants. The LGA has 14 public health facilities, four of which are focal sites. An average of three health workers works in each health facility. Previous records of OPV coverage from the state revealed that 96% of children 6-35 months of age had received 3 or more doses of routine OPV doses.

**Field´s work preparation:** on 7^th^ of October, 2015, the Osun state ministry of health was notified of an isolate of VDPV by the National Emergency Operating Centre (EOC), from an AFP case previously investigated through the conventional AFP surveillance system in the state. The state authority and partners were alerted immediately and a detailed investigation commenced on that same day by the state team, including the state Disease Surveillance and Notification Officer (DSNO). The LGA data on routine immunisation (RI), supplemental immunisation activities (SIAs) and surveillance were obtained from the LGA DSNO and the Local Immunization Officer (LIO) by the team. To improve the timeliness of the investigation, three sub-groups were formed from the state team, two of the sub-groups went to conduct household survey and stool sample collection from contacts and community, while the remaining group went for case investigation and surveillance desk review.

**Isolation and characterisation of poliovirus isolates:** the stool sample of the case was collected on 8 and 9, October 2015, while that of contacts (15 samples) and non-contact (40) were also collected subsequently each and sent to the laboratory for confirmation. Stool specimens were transported by the DSNO from the LGA to the national polio laboratory for poliovirus isolation in Ibadan, Oyo state. The collection and transportation of specimens followed the standard reversed cold chain system for AFP as previously described [6]. Viral isolation was performed on L20B and RD cell cultures and viral isolates were primarily identified by micro-neutralisation tests using poliovirus type-specific rabbit polyclonal antiserum (National Institute for Public Health and the Environment, Bilthoven, the Netherlands). Intratypic differentiation (ITD), VP1 sequencing and other necessary laboratory tests were conducted for determining poliovirus origin (wild or vaccine-related) [12]. The ITD was performed by polymerase chain reaction (PCR) restriction fragment length polymorphism (RFLP), real-time reverse transcription PCR and by enzyme-linked immunosorbent assay (ELISA). All tests were processed according to the standard guidelines recommended by WHO [13].

**Public health response:** a mop-up immunisation response was conducted on 9 November 2015 in Moore ward of Ife East LGA, where the polio event case lived. A total of 60 LGA healthcare workers went to house-to-house in groups of 3 (one vaccinator, one recorder and one mobilizer). All houses, schools, markets and busy streets in all settlements in Moore ward were visited. There were 15 senior supervisors from the state level who supervised the process along with partners (WHO, UNICEF and NPHCDA). Following the mop-up response in the affected ward, a three large-scale outbreak response followed in the whole of Osun state and adjourning states of Ekiti and Ondo. Two bordering LGAs of Ogun state were covered in the response. Activities conducted during the outbreak response included, vaccination of children 0-59 months with tOPV, collection of 15 stool samples from contacts and 40 stool samples from non-contacts for laboratory investigation. Others were collection of data on OPV coverage using the house-to-house data tools, including the tally sheet and training of 64 medical students/student nurses for the conduction of retroactive case searches in the affected ward of the LGA. The implementation was done in 64 settlements containing 1790 households. Intensified active case searches for AFP cases in the state by DSNOs, assistant DSNOs, healthcare workers, focal persons and community informants were also conducted.

**Social mobilization:** letters were written to schools and churches to inform them of the programme and the state and LGA made announcements on radio. Also, a radio documentary programme on immunisation took place with the state director primary health care and social mobilization officer.

**Data collection:** the caregiver of the case-patient served as informants during a detailed case investigation using the specified form in the AFP guideline. Data on immunisation history of under-five children were also obtained from caregivers in households in the immediate environment of the case-patient using a house-to-house data tool (Tally sheet). The OPV coverage data from 2012 to 2015 was obtained from the administrative reporting routine immunisation for the state, coverage surveys and immunisation histories collected during the investigation of AFP cases. Data on vaccination coverage among the under-five children investigated and records of OPV coverage between 2012 and 2015 were analysed using the Statistical Package for Social Sciences (SPSS) version 20. Frequency tables and chart were used to present the results.

**Description of the case-patient and the environment in Ife East LGA:** the case-patient was a one-month-old female who was the third child of her family. The child lived with parents and two other siblings in a multi-family structure. The travel history of the child revealed that she had lived in the same household since birth and no member of the family has travelled or received any visitors in 30 days before the onset of illness. The environment in which the child resides was filthy. Refuse dump was directly in front of the house. The entire fairly congested house was using a single pit latrine and a communal well in front of the house served as the primary source of water for the family.

**Clinical symptoms:** the child presented with several diverse clinical symptoms ranging from fever, boils on the hands, inability to move both arms, severe intestinal pains and death. Clinical symptoms as stated by primary caregivers suggested intestinal obstruction and infectious disease of the soft tissue of the upper limbs, resulting in septicaemia/osteomyelitis, causing weakness and inability to move the upper limbs.

**Delivery and vaccination history of child:** the child was delivered in a mission house in the area; ante-natal period and birth were uneventful. The routine immunisation card of the child showed that she had received two doses of OPV (at birth and first dose). Hence she was adequately immunised for her age. However, the child did not receive any dose of OPV during Supplementary Immunization Activity (SIA) since she was delivered after the last National Immunization Plus Days (NIPDs) implemented in the state.

**Laboratory:** the result of the stool samples sent for laboratory confirmation revealed that the patient was positive for the Vaccine Derived Polio Virus (VDPV) while other specimens including those of the contacts and non-contact collected were negative for any type poliovirus.

**Figure 2:**
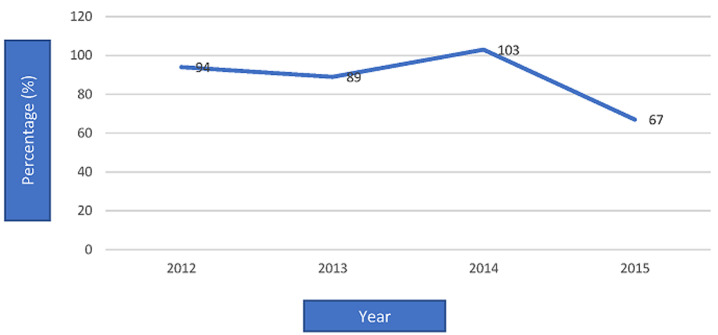
annual OPV 3 coverage, routine immunisation, Ife East LGA, Osun state 2012-2015

**Coverage survey:** Table 1 shows the outcome of the investigation on vaccination coverage among under-five children in households in the immediate environment of the case-patient. The majority (85.4%) of the children surveyed were 5-59 months and 62.5% were female. A high proportion (87.5%) of the children had received 3 or more doses of OPV, while 77.1% and 75% had been immunised during the last SIAs round and that before it respectively. More than two-thirds of the children were immunised with IPV. Record of OPV 3 coverage in the LGA in the last four years showed that the peak coverage of the vaccine was in 2014 (103%) however, there was a reduction in coverage in 2015 (Figure 2).

**Table 1:** outcome of vaccination coverage among households in the immediate environment of the case-patient

Variables	Frequency (n=48)	Percentage (%)
**Age (months)**		
<6	7	14.6
6-59	41	85.4
**Gender**		
Male	18	37.5
Female	30	62.5
**Routine OPV doses**		
0 dose	2	4.2
1 dose	3	6.3
2 doses	1	2.0
3 and above doses	42	87.5
**Immunised in last round**		
Yes	36	75.0
No	12	25.0
**Immunised in the prior round**		
Yes	37	77.1
No	11	22.9
**Immunised with IPV**		
Yes	1	14.3
No	6	85.7

**Impact of public health response:** in total, 5478 children 0-59 months were vaccinated in the Moore ward during the mop-up response, out of which, 2287 (41.7%) were vaccinated house-to-house, 2990 (54.6%) in schools while 201 (3.7%) in other places, including markets and busy roads. As a result of the retroactive case searches, one missed case of AFP was seen and investigated. The immunisation large-scale response using tOPV commences 14 November 2015, eight days after notification of the case was sent to the state team. In total, 3,296,998 children were vaccinated during the period, while 1,085,395 were vaccinated during the first round with coverage rate of 113.9%, 1,097,958 (coverage rate: 115.4%) during second and 1,113,645 (coverage rate: 117%) in third round.

Polioviruses; wild and vaccine-derived, have been reported more in the northern states compared to the southern part of Nigeria with a record of active immunisation activities [4,14]. The present case of VDPV in a southern state was the first of its kind to our knowledge, being a female and a one-month-day-old child. Majority of cases in the previous outbreaks were male children with an average age of two years [10,11]. Poliomyelitis is a disease of children, but all ages can be affected and there is no sex predilection [6,12]. The clinical and epidemiological characteristics of the case reported are consistent with what was reported in the literature [6,10,12]. Available clinical data from this report showed that the confirmed case had various symptoms, including the inability to move the affected limbs, fever and severe pains in the intestine, with the case ending up being dead. This finding is consistent with previous reports of cVDPV1 [10] and cVDPV2 infections [4], for which a cumulative 74% of cVDPV-associated AFP cases involved residual paralysis and fever typical of poliomyelitis. However, outcome data are not usually reported with the outbreaks [8,15]. The final classification of the virus is still in doubt and we recognised this as a limitation of our investigation. The laboratory report we have access to did not provide the gene bank assession number as well as the serotype of the virus. If we consider that it is an iVDPV, the immunological status of the infant is not documented regarding immune pathologies or immune deficiencies and no blood sample was collected for testing blood immunoglobulins (Ig) levels.

WHO recommends clinical and biological investigations, including diagnosis of immune deficiencies before classifying a VDPV as iVDPV [7,9]. More so, the vaccination profile of the case indicated that she was adequately vaccinated for her age, although she did not receive any dose of OPV during SIAs. Furthermore, there was no evidence of person-to-person transmission in the community to classify it as cVDPV. The outcome of the investigation on vaccination coverage conducted among the under-five children in the immediate environment of the case revealed that majority of them were vaccinated both in the last SIAs round and the round before it. Different from this report, earlier authors have attributed under-vaccination among children with the emergence of poliovirus infection. For instance, Kamadjeu *et al*. [16] observed that 55% of all WPV cases had never received OPV (so-called zero-dose individuals) and almost 80% were under-vaccinated (i.e. they received ≤3 doses of OPV) in Somalia. In addition, most patients with cVDPV2 and WPV were under-vaccinated in Nigeria [10]. However, at the population level, the record of administrative OPV coverage in the LGA in the last four years revealed a drop in RI from 94% in 2012, 83% in 2013, 103% in 2014 to 67% in 2015. This drop in coverage rate, if it is real, may have contributed to the re-emergence of the infection in the LGA which indicated a population immunity gap that enables the development of the poliovirus. This finding alone is in accord with previous studies suggesting that the principal risk factor for VDPV emergence and spread is low population immunity.

Other authors [4,7,17] reached similar conclusions using NP-AFP and NICS 2006 data to estimate coverage with type 2-containing OPV. Moreover, asides the drop in the RI in the affected LGA, the environmental characteristics of the case-patient may also contribute towards the emergence of the infection. We observed that the case had lived in a filthy environment with poor toilet and water facilities, which could aid the occurrence of the infection. Similar observations were also made in earlier reports [8,13]. The immunisation response administrative data revealed that a substantial number of children were vaccinated in the affected ward while more than 100% coverage rates were achieved during the three rounds of the large-scale response in the states where they were conducted. It is expected that this will boost the herd immunity and prevent transmission. A similar response has been conducted in previous outbreaks in Nigeria with an encouraging outcome. For instance, after two rounds of tOPV SIAs conducted in affected northern states in Nigeria in May and August 2009, monthly incidence of cVDPV2 decreased sharply from 35 cases in 2009 to 0-3 cases during September 2009-June 2010 [9].

The SIAs, being a mass vaccination campaign strategy aimed to administer additional doses of oral poliovirus vaccine (OPV) to each child aged <5 years [16], regardless of their vaccination history, may have proven effective in reducing the incidence of VDPV. The strategy has also been mainly used in many countries and has contributed to the 99% global reduction in the incidence of paralytic poliomyelitis observed since 1988 lunch of the global polio eradication initiative [18,19]. We identified some crucial strengths in the public health response embarked upon during this polio event. The state and LGA team were committed and there was the timely arrival of training, logistics and social mobilisation funds. The national government and partner agencies adequately provided financial support and there was an excellent and efficient state cold chain system. Also, evening review meetings at state and LGAs helped in correcting gaps for the next day´s work and there was intensive supervision by state team and partners during training and implementation. However, some gaps, including the non-release of counterpart funding by the state and LGA to aid logistics activities, late arrival of vaccines to the state, inadequate micro plans, poor team performance in the filing of the data tools and ineffective social mobilization, especially to schools and churches, were noted.

## Conclusion

Finally, to prevent the occurrence of poliovirus event or outbreaks subsequently, efforts should be directed to, improving environmental hygiene in households and improving OPV and inactivated polio vaccine (IPV) coverage which reduces the risk of VDPV in subsequent routine and supplementary immunisation activities. Public enlightenment on the importance of proper environmental sanitation should be embarked upon. Environmental inspection may be reintroduced to educate community members. There is a need for the state and LGA authorities to ensure ownership of the SIA and RI program, by ensuring the provision of adequate materials and funds required, as well as the early arrival of vaccines and relevant tools during campaigns.
